# Supplementary material for: Machine learning uncovers the Pseudomonas syringae transcriptome in microbial communities and during infection
Source: mSystems. 2023 Aug 28;8(5):e00437-23. doi: 10.1128/msystems.00437-23 (PMC10654099; doi:10.1128/msystems.00437-23)
Supplement: Supplemental Material — Supplemental notes, figure caption, tables, and references. [file msystems.00437-23-s0005.docx]

# Supplementary Information

**Supplementary Notes:**

***Supplementary Note S1: Antibiotic resistance in P. syringae may be triggered by indole***

*C. violaceum* was found to upregulate the activities of the Resistance-1 and Resistance-2 iModulons, with Resistance-2 being notably upregulated. This indicates that the bacteria may produce exometabolites that cause an upregulation of antibiotic resistance-related genes in *P. syringae*. A signaling molecule known to increase antibiotic tolerance through the activation of efflux pumps in other *Pseudomonas* species is indole(1). Additionally, a previous study showed that indole-containing compounds upregulate TetR and MarR family transcriptional regulators (PSPTO_4302, PSPTO_3617, and PSPTO_3749) belonging to the Resistance-1 iModulon, and *cueR* (PSPTO_0749) in the Resistance-2 iModulon(2). Both *B. thailandensis* and *C. violaceum* produce 3-indole-propionic acid, which may trigger an indole-dependent antibiotic resistance response in *P. syringae,* which has previously been observed in *P. putida*(3).

***Supplementary Note S2: Pectin may serve as a carbon source to P. syringae during carbon starvation in the apoplast***

The Carbon Starvation-2 iModulon contains genes involved in the transport and metabolism of various sugars, including arabinose and galactose, which are components of pectin(4). Additionally, the iModulon also contains genes encoding oxidoreductases, which may possibly be involved in the reduction of galacturonic acid, a major component of pectin, to form galactose(5). Hence, the upregulation of the iModulon during PTI-induced carbon starvation may indicate that *P. syringae* begins to break down the cell wall of *A. thaliana* during PTI to utilize pectin as a carbon source.

**Supplementary Figures:**

***Supplementary Figure S1.*** *Genes in the Nitrogen iModulon represent various nitrogen sources used under nitrogen starvation.* ***a.*** *Nitrogen iModulon gene membership.* ***b.*** *Pre-induced PTI using chitosan and flg22 upregulates the Nitrogen iModulon (SA: salicylic acid; n = 12 for elicitor type ‘none’, n = 3 for elicitor types ‘DMSO’, ‘water’, ‘flg22’, and ‘SA’, n = 2 for elicitor type ‘chitin’).*

**Supplementary Tables:**

***Supplementary Table S1.*** *Baseline conditions selected for each project*

| **Project** | **Baseline Condition** |
| --- | --- |
| algU_regulon(6) | empty_vector |
| co_culture(3,7) | mono_12.5 |
| immunity_2(8) | WT_sid2 |
| immunity_in_vitro(9) | WT_MM |
| immunity_isolated(9) | WT_WT |
| immunity_no_isolation(9) | WT_WT_mock |
| immunity_time(10) | mock_1hpi |

***Supplementary Table S2.*** *n values for error bars in Figure 2e-i*

| **Coculture condition** | **Time point (h)** | **n value** |
| --- | --- | --- |
| *P. syringae* | 12.5 | 4 |
| *P. syringae* | 25 | 4 |
| *P. syringae* | 30 | 2 |
| *P. syringae* | 35 | 4 |
| *P. syringae* | 40 | 4 |
| *P. syringae* | 45 | 4 |
| *P. syringae + C. violaceum* | 12.5 | 4 |
| *P. syringae + C. violaceum* | 25 | 4 |
| *P. syringae + C. violaceum* | 30 | 3 |
| *P. syringae + C. violaceum* | 35 | 4 |
| *P. syringae + C. violaceum* | 40 | 4 |
| *P. syringae + C. violaceum* | 45 | 2 |
| *P. syringae + B. thailandensis* | 12.5 | 3 |
| *P. syringae + B. thailandensis* | 25 | 3 |
| *P. syringae + B. thailandensis* | 30 | 4 |
| *P. syringae + B. thailandensis* | 35 | 4 |
| *P. syringae + B. thailandensis* | 40 | 3 |
| *P. syringae + B. thailandensis* | 45 | 3 |
| *P. syringae + C. violaceum + B. thailandensis* | 12.5 | 2 |
| *P. syringae + C. violaceum + B. thailandensis* | 25 | 1 |
| *P. syringae + C. violaceum + B. thailandensis* | 30 | 4 |
| *P. syringae + C. violaceum + B. thailandensis* | 35 | 4 |
| *P. syringae + C. violaceum + B. thailandensis* | 40 | 3 |
| *P. syringae + C. violaceum + B. thailandensis* | 45 | 4 |

***Supplementary Table S3.*** *Description of samples in Figure 3.*

| **Sample** | **Bacterial Strain** | **Host Genotype** | **Infiltration Agent** |
| --- | --- | --- | --- |
| WT_WT | *P. syringae* pv *tomato* DC3000 | *A. thaliana* wild-type Col-0 | none |
| WT_WT_mock | *P. syringae* pv *tomato* DC3000 | *A. thaliana* wild-type Col-0 | water |
| WT_WT_SA | *P. syringae* pv *tomato* DC3000 | *A. thaliana* wild-type Col-0 | salicylic acid |
| WT_WT_chitin | *P. syringae* pv *tomato* DC3000 | *A. thaliana* wild-type Col-0 | chitin |
| AvrRpt2_WT | *P. syringae* pv *tomato* DC3000 ectopically expressing AvrRpt2 | *A. thaliana* wild-type Col-0 | none |
| AvrRpt2_stp | *P. syringae* pv *tomato* DC3000 ectopically expressing AvrRpt2 | *A. thaliana stp1 stp13* mutant | none |
| AvrRpt2_b2b3 | *P. syringae* pv *tomato* DC3000 ectopically expressing AvrRpt2 | *A. thaliana cyp79b2 cyp79b3* mutant | none |
| AvrRpt2_DE | *P. syringae* pv *tomato* DC3000 ectopically expressing AvrRpt2 | *A. thaliana dde2 ein2* mutant | none |
| AvrRpt2_pad4 | *P. syringae* pv *tomato* DC3000 ectopically expressing AvrRpt2 | *A. thaliana pad4* mutant | none |
| AvrRpt2_sid2 | *P. syringae* pv *tomato* DC3000 ectopically expressing AvrRpt2 | *A. thaliana sid2* mutant | none |
| AvrRpt2_npr1 | *P. syringae* pv *tomato* DC3000 ectopically expressing AvrRpt2 | *A. thaliana npr1* mutant | none |
| AvrRpt2_PS | *P. syringae* pv *tomato* DC3000 ectopically expressing AvrRpt2 | *A. thaliana pad4 sid2* mutant | none |

**Supplementary References:**

[1. Molina-Santiago C, Daddaoua A, Fillet S, Duque E, Ramos J-L. 2014. Interspecies signalling: *Pseudomonas putida* efflux pump TtgGHI is activated by indole to increase antibiotic resistance. Environ Microbiol 16:1267–1281.](https://www.zotero.org/google-docs/?broken=2MFjrT)

[2. Han J-T, Li D-Y, Zhang M-Y, Yu X-Q, Jia X-X, Xu H, Yan X, Jia W-J, Niu S, Kempher ML, Tao X, He Y-X. 2021. EmhR is an indole-sensing transcriptional regulator responsible for the indole-induced antibiotic tolerance in *Pseudomonas fluorescens*. Environ Microbiol 23:2054–2069.](https://www.zotero.org/google-docs/?broken=EKQcic)

[3. Chodkowski JL, Shade A. 2020. Exometabolite dynamics over stationary phase reveal strain-specific responses. mSystems 5:e00493-20.](https://www.zotero.org/google-docs/?broken=8X3r08)

[4. Ropartz D, Ralet M-C. 2020. Pectin Structure, p. 17–36. *In* Kontogiorgos, V (ed.), Pectin: technological and physiological properties. Springer International Publishing, Cham.](https://www.zotero.org/google-docs/?broken=nCo2rl)

[5. D-galactopyranuronic acid (CHEBI:4153). https://www.ebi.ac.uk/chebi/searchId.do?chebiId=CHEBI:4153. Retrieved 3 February 2023.](https://www.zotero.org/google-docs/?broken=5A7c7j)

[6. Markel E, Stodghill P, Bao Z, Myers CR, Swingle B. 2016. AlgU controls expression of virulence genes in *Pseudomonas syringae* pv. *tomato* DC3000. J Bacteriol 198:2330–2344.](https://www.zotero.org/google-docs/?broken=XwcOpa)

[7.](https://www.zotero.org/google-docs/?broken=yXRrGx) Chodkowski JL, Shade A. 2022. Bioactive exometabolites drive maintenance competition in simple bacterial communities. bioRxiv https://doi.org/10.1101/2021.09.05.459016.

[8. Nobori T, Wang Y, Wu J, Stolze SC, Tsuda Y, Finkemeier I, Nakagami H, Tsuda K. 2020. Multidimensional gene regulatory landscape of a bacterial pathogen in plants. 7. Nat Plants 6:883–896.](https://www.zotero.org/google-docs/?broken=dpUmRb)

[9. Nobori T, Velásquez AC, Wu J, Kvitko BH, Kremer JM, Wang Y, He SY, Tsuda K. 2018. Transcriptome landscape of a bacterial pathogen under plant immunity. Proc Natl Acad Sci 115:E3055–E3064.](https://www.zotero.org/google-docs/?broken=yr6Yyz)

[10. Lovelace AH, Smith A, Kvitko BH. 2018. Pattern-triggered immunity alters the transcriptional regulation of virulence-associated genes and induces the sulfur starvation response in *Pseudomonas syringae* pv. *tomato* DC3000. Mol Plant-Microbe Interactions® 31:750–765.](https://www.zotero.org/google-docs/?broken=uKRnCH)
